# Increased Sensitivity of Computed Tomography Scan for Neoplastic Tissues Using the Extracellular Vesicle Formulation of the Contrast Agent Iohexol

**DOI:** 10.3390/pharmaceutics14122766

**Published:** 2022-12-10

**Authors:** Simona Vincenti, Alessandro Villa, Daniela Crescenti, Elisabetta Crippa, Electra Brunialti, Fereshteh Shojaei-Ghahrizjani, Nicoletta Rizzi, Monica Rebecchi, Michele Dei Cas, Angelo Del Sole, Rita Paroni, Vincenzo Mazzaferro, Paolo Ciana

**Affiliations:** 1 Department of Clinical Veterinary Medicine, Vetsuisse Faculty, University of Bern, 3012 Bern, Switzerland; 2 Department of Health Sciences, University of Milan, 20142 Milan, Italy; 3 Department of Pharmacological and Biomolecular Sciences, University of Milan, 20133 Milan, Italy; 4 Department of Oncology and Hemato-Oncology, University of Milan, 20122 Milan, Italy; 5 HPB Surgery and Liver Transplantation, Istituto Nazionale Tumori IRCCS Foundation (INT), 20133 Milan, Italy

**Keywords:** tumor boundaries, extracellular vesicles, iohexol, neoplastic cells, homing

## Abstract

Computed tomography (CT) is a diagnostic medical imaging modality commonly used to detect disease and injury. Contrast agents containing iodine, such as iohexol, are frequently used in CT examinations to more clearly differentiate anatomic structures and to detect and characterize abnormalities, including tumors. However, these contrast agents do not have a specific tropism for cancer cells, so the ability to detect tumors is severely limited by the degree of vascularization of the tumor itself. Identifying delivery systems allowing enrichment of contrast agents at the tumor site would increase the sensitivity of detection of tumors and metastases, potentially in organs that are normally inaccessible to contrast agents, such as the CNS. Recent work from our laboratory has identified cancer patient-derived extracellular vesicles (PDEVs) as effective delivery vehicles for targeting diagnostic drugs to patients’ tumors. Based on this premise, we explored the possibility of introducing iohexol into PDEVs for targeted delivery to neoplastic tissue. Here, we provide preclinical proof-of-principle for the tumor-targeting ability of iohexol-loaded PDEVs, which resulted in an impressive accumulation of the contrast agent selectively into the neoplastic tissue, significantly improving the ability of the contrast agent to delineate tumor boundaries.

## 1. Introduction

A major stumbling block in clinical oncology is the accurate determination of tumor boundaries during the diagnostic, therapeutic, and follow-up phases of cancer treatment. Indeed, cell-specific delineation of the extent of a tumor mass and its metastases, including micrometastasis detection, would allow clinicians to plan an extremely precise and patient-specific therapeutic approach. In a clinical setting, the main drawback of radiological contrast media is the lack of specificity toward the tumor tissue. Among the most commonly used strategies to overcome this limitation, specific radiopharmaceuticals are commonly used in clinical practice, but the limited spatial resolution of PET and SPECT scanners may reduce the detectability of smaller lesions and metastases. The targeted delivery of contrast agents to the neoplastic tissue has the potential to overcome these limitations [1]. Although several tumor-targeting tools have been proposed for the cancer-specific delivery of contrast agents, most of them have not reached clinical application yet; thus, efficient detection of tumor margins remains an important unmet medical need [2,3,4,5]. In the last decade, tumor-derived extracellular vesicles (EVs) have been proposed as biocompatible nanoparticles for the targeted delivery of therapeutic [6,7,8,9,10,11] and diagnostic [12,13,14,15,16,17,18] agents selectively to the neoplastic tissue. EVs is a general term indicating a mixed population of lipid nanoparticles containing protein, nucleic acids, and metabolites produced by virtually all cells of the organism and playing a key role in the intercellular communication among tissues, thus contributing to several physiological and pathological processes [19]. The growing interest in EVs as nanodelivery tools was raised by several works outlining many positive features of these nanoparticles including favorable pharmacokinetics (they do not accumulate in the body) [20,21], limited toxicity and immunogenicity [22], and the ability to cross the blood–brain barrier [23]. EVs can be loaded with a wide variety of molecules (from small molecules to entire viruses) and protect their cargo from degradation [8,24,25,26,27] until its selective delivery to the cancer tissue [28,29], thus reducing the potential side effects due to systemic administrations of toxic therapies [29,30]. Recent work from our laboratory demonstrated the feasibility of an autologous protocol for diagnostic intraoperative imaging based on patient-derived EVs (PDEVs) and used to selectively deliver a contrast agent to the tumor of the same patient from which EVs were isolated [18,29]. This study opened the way to loading EVs with contrast agents that would increase the detection sensitivity of different imaging modalities, thereby helping clinicians to obtain crucial information needed for patient therapy and follow-up, including the accurate localization of the primary and potentially metastatic neoplastic disease before and during surgery, and a precise measure of tumor reduction after therapy [31]. CT and positron emission tomography (PET) in conjunction with CT scans (PET-CT) are standard techniques used in clinical oncology to establish the diagnosis, assess the extent of neoplastic disease, plan appropriate therapy, and perform follow-up [31]. Iohexol is a safe, radiopaque contrast agent used worldwide for contrast-enhanced CT and PET-CT and is usually administered intravenously during the imaging procedure [32]. However, this contrast agent has no specific tropism toward tumor cells, and its ability to enhance the presence and extent of a tumor during imaging is strictly limited by the degree of vascularization of the tumor itself [33,34]. For this reason, iohexol shows contrast enhancement in highly vascularized structures without distinguishing between neoplastic and non-neoplastic tissue. If the neoplastic process does not have strong neovascularization, iohexol distributed through the blood vessels will not be able to efficiently diffuse into the tumor and highlight and delineate the tumor margins, thus, inadequately reflecting the extent of the neoplastic tissue [34]. Furthermore, being a small-molecule contrast agent, iohexol tends to be quickly eliminated from the body [32], making the time window for X-ray-based imaging very narrow. In the past decade, nanoparticles (NPs) have been tested as CT contrast agents with the intent of overcoming the abovementioned limitations of iodinated small molecular contrast agents [35,36]. In particular, a few studies focused on creating iohexol-integrated nanoparticles, which exhibited protracted retention within the tumor bed, increase in CT contrast, and longer circulation time [37,38,39]. However, to the best of our knowledge, no study has so far evaluated the iohexol formulation with EVs, although their biocompatibility and tumor-targeting properties, as well as their ability to selective delivering their content to the neoplastic tissue, make them particularly attractive to obtain a stronger and longer-lasting contrast signal for the iohexol CT. We here provide a preclinical proof-of-principle of a new imaging methodology based on the tumor-targeting ability of PDEVs that led to an impressive accumulation of iohexol selectively in the neoplastic tissue, thus greatly enhancing the ability of the contrast agent to delineate tumor boundaries.

## 2. Materials and Methods

### 2.1. Reagents

Regents were purchased from Sigma-Aldrich St. Louis, MO, USA if not otherwise specified.

### 2.2. EV Extraction from Blood of Colorectal Cancer (CRC) Patient

Venous blood (30 mL) was collected from three patients during preoperative analyses after approval by the Ethics Committee of the National Cancer Institute of Milan (Aut. INT 244/20). Blood was collected in EDTA-conditioned vials and immediately centrifuged at 1750× *g* for 10 min at room temperature to remove blood cells and prevent platelet activation and the release of platelet-derived EVs. Supernatants were transferred to new tubes (the bottom 10% of supernatant above the blood cells was discarded), and samples were centrifuged again at 3000× *g* for 10 min at room temperature. Supernatants were collected and processed by ultracentrifugation for 2 h at 100,000× *g* at 4 °C in an Optima L-80 XP ultracentrifuge (Beckman Coulter, Indianapolis, IN, USA) with an SW32Ti rotor (Beckman Coulter). Supernatants were aspirated and the EV-containing pellets were resuspended in 100 μL phosphate-buffered saline (DPBS, EMD Millipore, Burlington, MA, USA) and stored at −80 °C until use.

### 2.3. Size Distribution Determination by Nanoparticle Tracking Analysis (NTA)

The size distribution and concentration of EV, EV-ICG, and EV-iohexol formulations were analyzed by Nanoparticle Tracking Analysis (NTA) using a Nanosight model NS300 (Malvern Panalytical Ltd, Malvern, UK) NTA with a blue (404 nm, 70 mV) laser and sCMOS camera. NTA was performed for each sample by recording three 90 s videos, which were subsequently analyzed using NTA software 3.0 (Nanosight). The detection threshold was set to level 5 and the camera threshold to level 15.

### 2.4. Cryo-Electron Microscopy (EM)

Cryo-EM images (150 fields) were acquired with an FEI Talos Arctica 200 kV FEG electron microscope equipped with an FEI Falcon 3EC direct electron detector and Volta Phase-plate. Prior to Cryo-EV imaging, the samples were vitrified on an FEI Vitrobot IV system and processed as previously reported.

### 2.5. Immunoblotting

For immunoblotting, extracellular vesicles were isolated from patients’ blood according to the protocol described above. After the ultracentrifugation step, the supernatants were removed, and the EV-containing pellets were resuspended in a proper volume of 1X RIPA buffer (150 mM NaCl; 1% NP-40; 0.5% sodium deoxycholate; 0.1% SDS; 50 mM Tris-HCl, pH 8.0) supplemented with protease inhibitor cocktail (Roche, Penzberg, Germany). EV protein concentrations were quantified using a Bradford assay kit (ThermoFischer Scientific, Waltham, MA, USA). Twenty micrograms of EV protein lysates were separated to 4–10% SDS-PAGE using beta-mercaptoethanol as the reducing agent and transferred to nitrocellulose membranes (Amersham Biosciences, Amersham, UK). The membranes were then blocked in 5% nonfat dry milk in TBS-T (0.2% Tween-20) at RT and incubated overnight with the primary antibody against exosomal TSG101 (4A10, 1:500, Abcam, Cambridge, MA, USA). Immunoreactive bands were visualized with chemiluminescence using the ECL Western Blotting Analysis System according to the manufacturer’s instructions (Amersham Biosciences, Amersham, UK).

### 2.6. Evaluation of EV Integrity

A total of 1 × 10^8^ PDEVs in 660 µL of DPBS were stained by adding 2 µL of 1mM carboxyfluorescein succinimidyl ester (CFSE), a membrane-permeant dye that releases fluorescence in the presence of EV esterases, to reach a final concentration of 3 µM CFSE, followed by incubation at 37 °C for 20 min. PDEV integrity was evaluated using a Cytek Aurora flow cytometer (Cytek Biosciences, Fremont, CA, USA). The cytometer was calibrated to detect the SSC at 405 nm with a sensitivity of 1000 arbitrary units. The laser at 488 nm was tuned to 70 mW to detect CFSE fluorescence. The green fluorescence was measured using a diode array detector. Reference beads were used to control the performance of the instrument. After 10–15 s of sample collecting to stabilize the flow rate, the volume of the recording was adjusted to 15 L and the flow rate was set to “Low.” The flow meter of the cytometer recorded the exact volume measured. The percentage of CFSE-stained PDEVs indicates the level of EV integrity in the preparation. DPBS enriched with CFSE and unstained EVs were employed as background controls.

### 2.7. EV Loading with Iohexol and Indocyanine Green (ICG)

Iohexol is a non-ionic, water-soluble molecule with lateral hydrophilic chains that could struggle to permeate into the EV membranes. For this reason, two techniques, passive diffusion, and sonication were evaluated to load EVs with iohexol.

Passive diffusion was carried out by adding 10^8^ EVs from MC-38 cells suspended in 50 µL of Dulbecco’s phosphate buffered saline (DPBS, EMD Millipore, Burlington, Massachusetts , United States) to 950 µL of iohexol (Accupaque Iohexol Injection 300 mg/mL, GE Healthcare, Milano, Italia). The mix was incubated for 16 h at 4 °C. Then, samples were brought to a volume of 16 mL with DPBS and ultracentrifuged at 100,000× *g* for 90 min at RT (Himac ultracentrifuge CP100NX with rotor P50AT2, Eppendorf Himac Technologies Co., Takeda, Japan). After ultracentrifugation, the supernatant was eliminated and the EV-containing pellets were resuspended in 100 µL DPBS.

Sonication of the EV suspension was performed by means of a sonicator (Sonicator Branson Sonifier 250, ThermoFischer Scientific, Waltham, MA, USA). Briefly, 10^8^ EVs from MC-38 cells suspended in 50 µL of DPBS were mixed with 950 µL of 300 mg/mL iohexol, then the mix was subjected to mild sonication with an amplitude of 20% for 6 cycles of 30 s each, followed by 2 min and 30 s of cooling down. After sonication, the solution was incubated at 37 °C for 1 h to allow EV reformation. To remove the excess iohexol, each sonicated preparation was brought to a volume of 16 mL with DPBS and ultracentrifuged at 100,000× *g* for 90 min at RT (Himac ultracentrifuge CP100NX with rotor P50AT2). Finally, supernatants were removed and the EV-containing pellets were resuspended in 100 µL DPBS.

ICG was loaded into PDEVs as previously described (Villa et al. 2021). Briefly, 10^8^ PDEVs suspended in 50 μL DPBS 150 μL were added to 150 μL of a water solution of 5mg/mL ICG (Sigma) and incubated for 16 h at 4 °C. Then, samples were centrifuged at 100,000× *g* for 90 min. After supernatant removal, pellets were resuspended in 150 μL of DPBS.

### 2.8. Animals

All animal experiments were approved by the Italian Ministry of Research and University (permission number: 214/2020) and regulated by a departmental panel of experts. C57BL/6NCrl (Charles River, MGI: 2683688) mice were maintained at the animal facility of the University of Milan under standard conditions according to institutional guidelines. After an acclimatization period of 14 days, murine syngeneic grafts were established by s.c. injections of 2 × 10^6^ MC-38 cells into the neck of 12-week-old male C57BL/6 mice. The health status of the mice in the experimentation was monitored daily, and as soon as signs of pain or distress were evident, the mice were euthanized. EV injections were performed when the tumor size reached a diameter of ~10 mm. For the homing tests, mice engrafted with tumors were i.v. injected with 2–4 × 10^9^ EVs/kg.

### 2.9. In Vivo and Ex Vivo Fluorescence Imaging

In vivo and ex vivo fluorescence imaging, sessions were carried out 24 h after EV treatment using a SPY Elite intraoperative imaging device (Stryker Italia, Roma, Italy), equipped with filters for near-infrared signal detection, following the manufacturer’s instructions. Mice were anaesthetized using isoflurane (Isoflurane-V et; Merial, Lyon, France) and kept under anesthesia during imaging sessions. For ex vivo imaging, mice were sacrificed by cervical dislocation. Immediately after death, selected organ imaging was also carried out.

### 2.10. In Vivo and Ex Vivo CT Scans

To investigate the presence or absence of iohexol in the explanted murine organs, we carried out a computer tomography analysis using a GE LightSpeed 16 (GE Healthcare, Chicago, IL, USA) CT scanner. The X-ray tube was operated at a voltage of 120 kVp with a tube current of 40 mA and an exposure time of 2 s and a slice interval of 0.48 mm. Three-dimensional reconstruction of the in vivo and ex vivo scans was rendered using the 3D-viewer plugin of ImageJ software (NIH). Densitometric measures were obtained using the Image Measure function of ImageJ (NIH).

### 2.11. Quantification of Iohexol Incorporated into EVs and in Tissue Homogenates

Quantification of iohexol was performed by both high-performance liquid chromatography (HPLC) coupled with a UV detector (LC-UV) and high-resolution liquid chromatography coupled with a mass spectrometer (LC-HRMS/MS). For quantification by HPLC, an HPLC with a UV detector (mod. 1000) and autoprobing (mod. 4000) was used with a Phenomenex, Bondclone 10 µm C18, 300 × 3.9 as the column. The flow rate was 0.8 mL/min with a UV wavelength of 250 nm and an injection volume of 10 µL. Acetaminophen was used as an internal standard (IS) because its spectrochromatographic molecular properties are similar to those of iohexol. For the analysis, a solution of 1 mg/mL acetaminophen in ethanol was prepared. This solution was then diluted with water to give a concentration of 25 μg/mL. The iohexol (Accupaque Iohexol Injection 300 mg/mL) was titrated in iodine at a concentration of 300 mg/mL, which corresponds to 647 mg/mL iohexol. To establish the calibration line, an initial dilution was made from the iohexol stock solution to obtain a final concentration of 100 mg/mL iohexol. This was followed by a 1:100 dilution and finally serial 1:2 dilutions. Vials for the calibration line were prepared by adding 10 μL internal standard (25 μg/mL) to 10 μL standard solutions and 180 μL water, resulting in a linearity range of 50 μg/mL to 0.163 μg/mL iohexol. Iohexol quantification was also carried out on tissue homogenates. Briefly, tissues were weighed, inserted in a 2 mL vial containing a stainless-steel sphere and 1 mL of MilliQ water, shaken for five cycles of 30 s at a speed of 30 shakes/second, then centrifuged at 16,000 rcf to pellet tissue debris; finally, supernatants were collected for HPLC/MS analysis. To prepare the samples, 40 μL of methanol (1:5 dilution) was added to 10 μL of sample, followed by sonication and centrifugation to precipitate the proteins present. Finally, 10 μL of the supernatant was collected and 90 μL of water (1:10 dilution) was added. In this way, the total dilution of the initial sample was 1:50. The procedure for establishing the calibration line included: diluting Accupaque iohexol 300 mg/mL to obtain a 6 μM iohexol solution and performing a serial dilution to a concentration of 0.09 μM. The individual dilutions formed the points on the calibration line. Quantification of samples was performed using the Shimadzu UPLC instrument coupled to the Triple TOF 6600 Sciex (Sciex, Concord, Vaughan, ON, Canada) equipped with the Turbo Spray IonDrive. All samples were analyzed by electrospray ionization (ESI) in positive polarity (a mild ionization technique usually used for substances that are in solution in ionic form or are readily ionized). The analytical conditions were as follows: GS1 (nebulizer gas): 55, GS2 (drying gas): 65, CUR: 35, with a capillary voltage: 5.5kV, a temperature of 500 °C at the source, 45 °C in the column, a dusting potential of 70 eV, ionization energy: 70 ± 15 eV. The column was a reversed-phase Acquity HSS T3 C18 column 1.7 μm, 2.1 × 100 mm (Waters, Franklin, MA, USA) equipped with a precolumn; the mobile phase was: (A) water and (B) acetonitrile. Both contained 0.1% formic acid and had a flow rate of 0.4 mL/min. Under these conditions, iohexol has a retention time of 3 min and peaks at 821.9 *m*/*z*. Subsequent fragmentation of iohexol results in a higher peak at 374.98 *m*/*z*, which is used for quantification.

## 3. Results

### 3.1. Isolation and Characterization of PDEVs from Colon Cancer Patients

**Figure 1:**
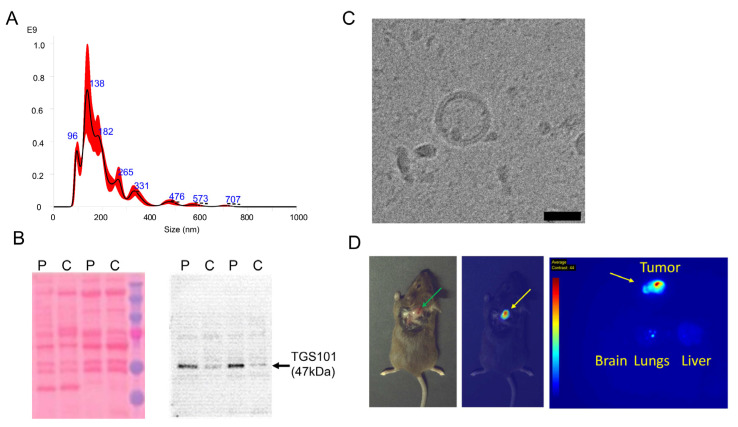
Characterization of EVs from CRC patient. The PDEVs displayed size distribution, shape, EV-specific marker expression, and tumor-homing capability comparable with the PDEVs used for the previous experiments. (**A**) Representative NTA analysis of particle size distribution of PDEVs. The red line represents the standard error of the mean. Details on the size distribution and concentration are given in Supplementary Table S1. (**B**) Immunoblot analysis of the Tumor Susceptibility Gene-101 (TSG101) expression in plasma-derived EVs from patients (P) or healthy controls (**C**). The uncropped blot is reported in Supplementary Figure S3. (**C**) Representative EV morphology and size obtained by acquiring 150 fields using cryo-electron microscopy, scale bar: 100 nm. More examples of vesicles imaged with cryo-electron microscopy are shown in Supplementary Figure S1. (**D**) Representative pictures obtained by imaging of the biodistribution of PDEVs loaded with the fluorescent molecule ICG, captured using the intraoperative imaging device Stryker SPY Elite. The intensity of the fluorescence signal is represented with pseudocolors: blue: low, yellow: medium, and red: high photon emission. The green arrow in the left panel shows the exposed tumor. The yellow arrows in the center and left panels show the fluorescent signal produced by the accumulation of PDEV-ICG in the tumor.

PDEVs were isolated from 30 mL of whole blood of patients affected by colorectal tumors and characterized according to the guidelines described in the ‘Minimal information for studies of extracellular vesicles (MISEV2018)’ documents [40]. In keeping with previous experiments [18], PDEVs had dimensions in the range of 100–400 nm (Figure 1A), expressed the specific biomarker TSG101 (Figure 1B), and displayed correct morphology and integrity as demonstrated by cryo-EM (Figure 1C, Supplementary Figure S1). PDEV integrity was evaluated by flow cytometry of CFSE-stained EVs, showing the integrity of ≈80% of particles (Supplementary Figure S2). To confirm the tumor selective tropism of our PDEV preparation, we followed a well-established protocol developed in our laboratory [18,29]. In brief, PDEVs loaded with a passive diffusion protocol with the fluorescent dye indocyanine green (PDEV-ICG) were used to test the homing ability of purified EVs; two groups of three C57BL/6 wild-type mice subcutaneously (in the neck area) implanted with the syngeneic MC-38 colorectal cancer cell line, were individually injected with 3 × 10^8^ PDEV-ICG or with 500 nmol free ICG as control, and biodistribution was assessed 24 h after injection in vivo and ex vivo by optical imaging using the intraoperative imaging device Stryker Spy Elite (Figure 1D). In line with our previous studies [18], only mice injected with ICG embedded in the PDEV preparation showed a specific fluorescent signal released by the neoplastic tissue.

### 3.2. Evaluation of Loading Methods to Incorporate Iohexol in EVs

**Figure 2:**
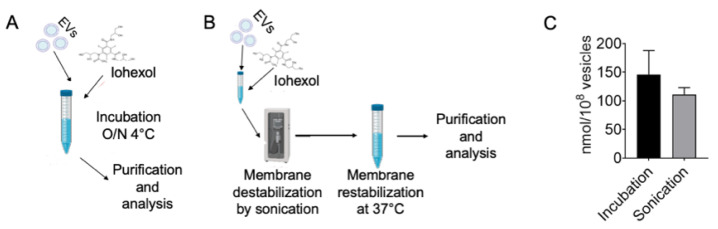
Loading strategies and evaluation of the iohexol incorporation in PDEVs. Schematic diagram of the loading protocols via passive diffusion (**A**) or sonication (**B**). (**C**) HPLC quantification of the iohexol incorporation in 10^8^ PDEVs with the two protocols. Bars represent mean and s.e.m.

Iohexol is a nonionic molecule with hydrophilic side groups, which are chemical-physical characteristics different from those of molecules that were previously loaded into EVs by our research group [11,18,29]. In order to find an effective strategy to introduce the molecules into the EVs, we started by comparing the efficiency of loading by passive diffusion (incubation 16 h at 4 °C) or forcing the incorporation by using sonication, as described in the Materials and Methods section and schematized in Figure 2A,B. The two methods had similar efficacy as both allowed the loading of an appropriate amount of iohexol in the PDEVs (Figure 2C). In particular, we determined by HPLC the average values of 98 nmol in the passive-loaded EVs versus 126 nmol in the sonicated incubated samples for 10^8^ PDEVs (Figure 2C). Since we felt that passive incubation was more convenient and simpler, we decided to adopt this loading method to produce the PDEV formulation (PDEV-IOX) for the following experiments.

### 3.3. PDEVs Loaded with Iohexol Selectively Target and Accumulate in Neoplastic Tissue in a Mouse Model of Colorectal Cancer

**Figure 3:**
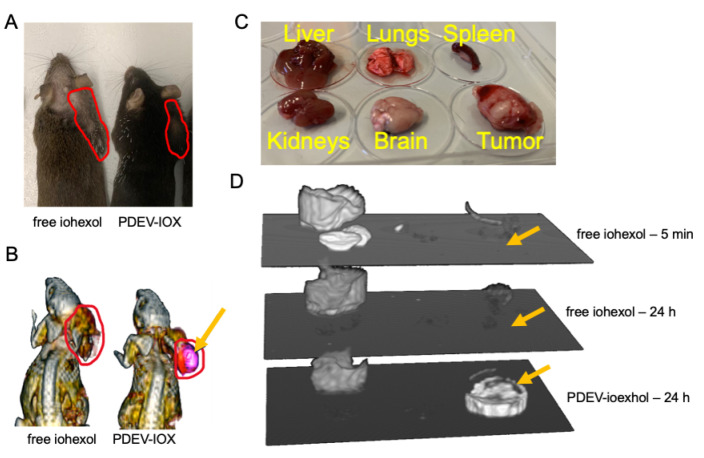
CT scans of the hyperintense area of tumor-bearing mice injected with different formulations of iohexol. (**A**) Photograph of representative mice showing the localization of the tumor (red line). (**B**) CT scans of the same mice with the hyperintense area (pink area indicated by the yellow arrow) showing the iohexol accumulation 24 h after treatment; localization of tumor is indicated with the red line. (**C**) Representation of the organ distribution in each 6-well plate used for the ex-vivo CT scan analysis of iohexol accumulation. (**D**) CT scans of three stacked plates containing the organs (from three individuals representative of the three groups where they belong) with the same position reported in (**C**); tumors are indicated by the yellow arrows. CT hyperintense signals in the tumors could be detected only 24 h after treatment with the PDEV-iohexol formulation.

Following preparation and characterization, we tested the homing ability of PDEVs loaded with iohexol in the same tumor model in which we previously assayed ICG-loaded EVs. Briefly, C57BL/6 wild-type mice were subcutaneously implanted (in the neck area) with the syngeneic MC-38 colorectal cancer cell line, and, once the tumor size reached a diameter of ~10 mm, two groups of three mice were i.v. injected with 3 × 10^8^ PDEV-IOX or with 2.2 µmol free iohexol in phosphate buffer as control—a dose in line with that used in clinical practice for diagnostic purposes. After 24 h, a time span sufficient for the complete wash-out of free iohexol, which has a biological half-life of 121 min, the mice were subjected to in vivo CT scans to evaluate the PDEV-driven accumulation of the molecule in the tumor tissue (Figure 3A,B). This imaging technique allows the detection of the accumulation of a radiopaque contrast agent such as iohexol, which results in a bone-like, hyperintense region. All mice treated with PDEV-IOX showed a strong hyperintensity of the area affected by the subcutaneous tumor (Figure 3B), while no specific signal from the tumor was detected in mice injected with free iohexol (Figure 3B), thus indicating that PDEVs promote the accumulation and stabilization of the contrast agent into the neoplastic tissue.

### 3.4. Ex Vivo Analysis with CT Scans

**Figure 4:**
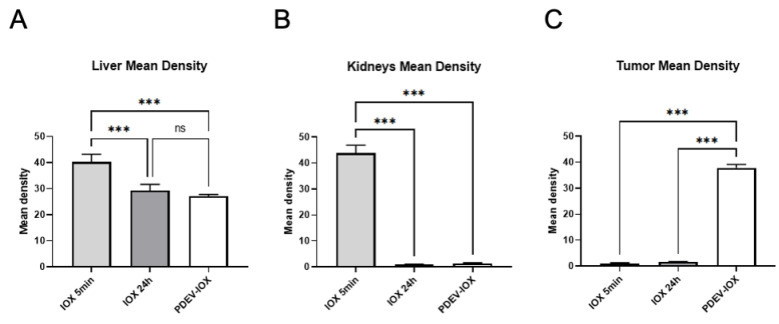
Semiquantitative densitometric analyses of CT scans of selected organs. Densitometry quantification of the signal of the CT-scan images for explanted liver (**A**), kidney (**B**), and tumor (**C**); ex vivo analysis was carried out for the organs explanted from mice after 5 min (IOX 5min) or 24 h (IOX 24 h) injection with free iohexol or 24 h after administration of a PDEV-formulated iohexol (PDEV-IOX). Bars represent mean and s.e.m.; ***: *p* < 0.01 by one-way ANOVA.

To better define the biodistribution of PDEV-IOX, we performed a series of ex vivo experiments on the mouse models of colorectal cancer with the purpose of evaluating the presence of iohexol in the organs involved in the distribution process of the drug (liver, lungs, spleen, kidneys), in the brain as a negative control (since iohexol molecules do not cross the blood–brain barrier), and in the tumor. To this end, one experimental group of n = 5 mice was i.v. injected with 10^9^ PDEV-IOX, and two control groups of n = 5 mice each with 2.2 µmol free iohexol. One of the control groups was sacrificed 5 min after treatment, a time span that allows the distribution of iohexol in the organism and corresponds to the standard protocol used in clinical practice. The mice from the PDEV-IOX group and those from the second control group were sacrificed 24 h after treatment. After sacrifice, tissues were explanted and snap-frozen immediately for further CT-imaging analysis. For the imaging sessions, tissues from each animal were placed in 6-well plates and cast for the comparative CT analysis (Figure 3C,D). The 3D-reconstruction pictures of the CT scan showed a clear iohexol-related hyperintensity of the tumor samples deriving from mice that received EV-IOX injection, while no hyperintensity was detected in the tumor samples deriving from the mice which received the injection of free-iohexol either 5 min or 24 h after injection (Figure 3D). The radiopacity of the liver was easily detectable in the 3D reconstruction in all animals showing a homogenous intensity independent of the received treatment; the radiopacity of the liver is a well-known phenomenon due to the density of liver parenchyma, which is usually greater than that of the other solid organs of the upper abdomen (spleen, kidneys, and pancreas) [41]. Then, to compare the signal intensities of the various organs, semiquantitative densitometric analysis was performed on CT images for all explanted organs (e.g., livers, kidneys, and tumors) (Figure 4A–C). The intensity of the signal from the liver was comparable in all the group components of the 24 h treatment, while it was slightly increased in the group treated with free iohexol for 5 min, suggesting that at this time point—as expected—iohexol diffused also in the liver, thus leading to the increased signal intensity. The signal detected in the kidneys—the excretion route of iohexol—was clearly detectable only in the control animals sacrificed 5 min after the injection of free iohexol, while in both groups treated for 24 h the signal disappeared, possibly due to the complete elimination of the contrast agent freely circulating in the bloodstream. In concordance with the CT-imaging data, tumoral tissues showed an increased density only in mice injected with PDEV-IOX and examined 24 h after injection; thus suggesting that PDEVs loaded with iohexol are able to accumulate in the tumor and that the PDEVs protect the incorporated molecule, increasing its half-life.

### 3.5. Quantification of Iohexol Incorporated into EVs in Tissue Homogenates

**Figure 5:**
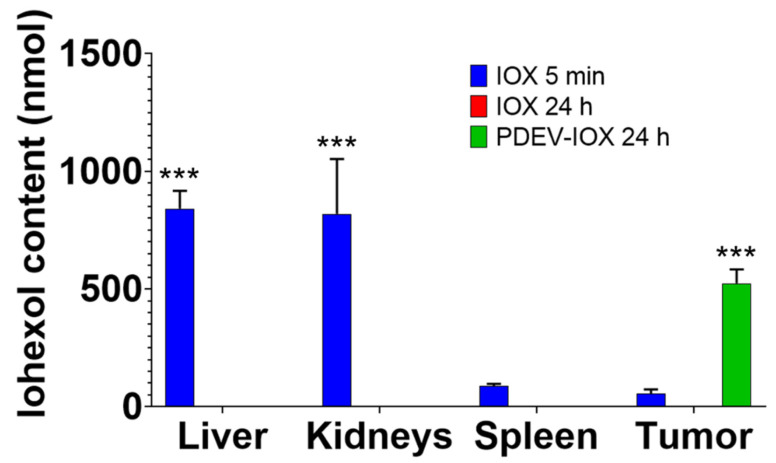
Quantification of iohexol accumulation in the organs of mice injected with different formulations of the contrast agent. HPLC-MS analyses of iohexol content in selected organs following injection with either short-time (5 min, blue bars), long-time (24 h), free iohexol (red bars), or PDEV-iohexol (green bars). Bars represent mean and s.e.m.; ***: *p* < 0.01 by two-way ANOVA.

Iohexol accumulation was quantified in homogenized tissues by HPLC-MS. To this end, a specific analytical protocol was developed to allow the chromatographic separation of iohexol from other components present in the biological matrix. This is necessary because, although the proteins were precipitated with methanol, it is inevitable that enzymes, lipids, and other substances may be released in the homogenate and contaminate the analyzed medium. The procedure included the choice of an appropriate HPLC column (Acquity HSS T3 C18 reversed-phase column), mobile phase composition, flow rate, and temperature. These changes allowed us to resolve the peak for iohexol, which had a retention time of 3.48 min. Using these analytical conditions, we were able to determine that iohexol was not degraded in the biological matrix and the retention time was unchanged compared to the standard molecule suspended in water. HPLC-MS analysis of the organs taken from mice treated with free iohexol showed that in the group sacrificed 5 min after the treatment, the presence of the contrast agent was mainly found in the liver and kidneys (organs involved in the metabolism and excretion of the contrast agent, Figure 5); in these organs, the contrast agent was no longer detectable in mice sacrificed 24 h after the injection of free iohexol (Figure 3D): this result was expected because it is known that > 90% of the free iohexol is physiologically excreted from the organism in the first 24 h [42]. The analysis also revealed that, when animals were sacrificed 5 min after the injection with free iohexol, a residual amount of the molecule (<6% of the total injected iohexol) was present also in the spleen and tumor, as was predictable given that these organs are highly vascularized (Figure 5). In contrast, analysis of the tumors revealed that a remarkable amount of iohexol (>500 nmol) was present only in the animals treated with PDEV-IOX, suggesting again that the specific tropism of the patient-derived EVs leads to the accumulation of the nanoparticles along with their cargo in the tumor. Interestingly, the mean content of iohexol measured in the tumor was >50% of the iohexol loaded into the PDEVs, thus providing evidence that more than half of the injected PDEVs targeted the tumor and accumulated there with their modified cargo.

## 4. Discussion

During the clinical management of a patient affected by a solid malignant tumor, the value of a CT scan in terms of detection of tumor boundaries and metastases is in several cases limited. This is due to the intrinsic poor radiopacity of the neoplastic tissue, which cannot be greatly improved by the administration of contrast agents. Iohexol, the most used radiopaque contrast agent worldwide, is administered intravenously and remains in the bloodstream until it is excreted in the urine; therefore, the poor accumulation observed in the neoplastic tissue is mainly dictated by the tumor vascularization. Some previous studies that tested nanoparticles with incorporated iohexol in X-ray-based imaging showed higher iohexol concentration and retaining time within the tumoral tissue in comparison to the free iohexol [37,38,39]. Concerning the retention time, these other studies described a CT contrast still visible at 4 h [39] and 6 h [38] post injection. Although the different methodology makes a comparison between these studies and our study difficult, we consider of important note the fact that, in our study, 24 h after injection a strong contrast signal within the tumoral tissue was still clearly detectable, despite the loading efficiency (12%) being lower than those calculated for the other nanostrategies. The longer duration of the contrast could have dramatic clinical applications, allowing clinicians to perform several, consecutive imaging studies with diagnostic and intraoperative purposes (i.e., CT and intraoperative fluoroscopy). Using this novel approach, we have delivered iohexol in the tumoral tissue reaching an extremely high (local) concentration, thus overcoming the limited ability of the compound to cross biological membranes. This is achieved because of the spontaneous tumor-targeting ability of PDEVs [18,29]; most importantly, the use of autologous material withdrawn from the same patient receiving the treatment overcomes the biocompatibility and toxicity problems inevitably linked with synthetic [43,44] or biological nanoparticles [45], hence facilitating the clinical translation of this iohexol formulation.

Though attractive for the abovementioned reasons, the extended use of PDEVs as a carrier of theranostics is limited by the loading process, which is largely unpredictable. For instance, the PDEV formulation of iohexol, a large (about 1000 daltons of molecular weight) hydrophilic molecule, was expected to require methodologies actively facilitating the membrane permeation; on the contrary, we were much surprised to observe that iohexol passively diffused through the EV membranes. This passive accumulation into EVs was puzzling and unexpected. Nevertheless, our HPLC-MS data clearly demonstrated that large quantities of iohexol were indeed sequestered by EVs (about 120 nmol/10^8^ EVs) demonstrating similar permeation efficacy for passive diffusion or electroporation methodologies (Figure 2). The molecular basis of this iohexol ability to be captured by EVs was not further investigated since it was not the focus of the current work, even if we may speculate that an active protein-mediated transport through the membrane, and/or a mechanical assembly/disassembly reaction of the EVs leading to the encapsulation of the medium component, and/or a stable interaction of the molecule with structures of the EV membrane (lipids or proteins) might be possible mechanisms of the iohexol uptake. Whatever the uptake mechanism is, the ability of PDEVs to deliver the cargo to the neoplastic tissue was unaffected by the presence of iohexol and led to the release of the contrast agent to the neoplastic tissue in living mice. After administration, in its journey to the tumor, the PDEV-incorporated iohexol was protected by the metabolism and excretion mechanisms that normally shorten to 2 h the contrast agent half-life in the body [46], which, instead, was widely prolonged by the PDEV-mediated accumulation into the neoplastic tissue. Indeed, 24 h after administration, the total amount of contrast agent delivered to the tumor was surprisingly high: about 50% of EVs-formulated iohexol (about 500 nmol in 10^9^ EVs) was selectively released into the neoplastic tissue, with virtually undetectable amounts of the molecule in other tissues, including the liver and kidneys, where the free iohexol is metabolized and excreted, respectively [46,47]. This high and selective accumulation of the contrast agent greatly increased the CT signal-to-noise ratio, thus opening new scenarios for the possible clinical applications of the CT scan with the PDEV-formulated iohexol. It is tempting to speculate that this protocol would magnify the CT sensitivity to the extent that is expected to provide a detailed definition of tumor margins and potentially the presence of normally undetectable metastases, which is particularly relevant for the generation of an ad hoc multimodal therapeutic plan. In fact, one of the biggest pitfalls encountered during therapeutic planning in oncology is the uncertainty around the severity of local invasion, as well as the regional and distant extension of the primary tumor [48,49]. One of the consequences of this is imprecise surgical planning, which can lead to an incomplete or inadequate neoplastic excision. Highly accurate margin detection pre- and intra-operatory could be extremely relevant to improving the ability of the surgeon to eradicate the disease; for example, for the primary or metastatic tumors of the gastrointestinal tract where the correct surgical intervention can contribute to the total eradication of the disease [50,51,52], or for glioblastoma tumors, which are phenotypically multiform with several prolongations inside the brain parenchyma whose margins are difficult to be mapped before and during the intervention [49].

Once the clinical applicability of the PDEVs-based autologous protocol with diagnostic molecules has been demonstrated, the high local concentration of the delivered cargo would be suitable for therapeutics or theranostic purposes. Bew EV formulations of small molecules or biologics [53] are expected to magnify the efficacy of these drugs, while significantly limiting their systemic toxicity [11], thus creating a novel unexplored condition for already approved anti-neoplastic therapies—a local concentration that would not be reachable with the standard systemic administration.

As previously stated, the use of autologous PDEV formulations [11] will greatly facilitate their translation into the clinical setting; in particular, PDEVs-iohexol formulations can be obtained with passive diffusion as the loading mechanism and with a purification methodology based on ultracentrifugation. These few simple steps can be easily integrated into the current surgery or therapeutic protocols for many different types of cancer patients. Indeed, the EV homing ability was demonstrated for several different solid tumors, including mammary, lung, colorectal, and prostatic cancers [54,55,56], therefore widening the spectrum of the potential applications of autologous PDEVs.

Current issues that restrict the clinical translation of autologous PDEVs for the delivery of theranostics are the limited knowledge of their homing mechanism and the consequent establishment of adequate quality control ensuring the reproducibility and safety of batches derived from different patients. In terms of quality control, it is of utmost importance to perform a risk analysis to determine the key parameters that need to be monitored to ensure reproducibility during production, isolation of EVs, and their subsequent loading with contrast agents. Indeed, the technical challenges and difficulty in standardizing the processes of isolation, quantification, and characterization are barriers to their clinical use. Although challenging, the quality-control issues could be overcome by the natural reproducibility of the PDEV preparation from oncological patients, which, in our experience, seems to be very constant in terms of number and homing ability. This is surprising considering the potential heterogenicity of the number of tumor-derived EVs inside the mixed population circulating in the blood of oncology patients. Although not yet experimentally confirmed, our current hypothesis is that tumor-derived EVs may be positively selected in the patient due to their longer half-life [57,58], likely a consequence of their ability to escape from metabolic degradation. This would lead to a predominant population of tumor-derived EVs circulating in the blood, which may explain, at least in part, their constant number and similar homing efficacy even in different types of patients.

## 5. Conclusions

The use of an appropriate combination of biomarkers and imaging techniques will become standard practice in the future. As cancer incidence and mortality rates increase, it is imperative to explore new approaches for the early detection and accurate characterization of tumors. EVs have great potential for diagnosis and targeted therapy. With this work, we have demonstrated that the generation of EVs loaded with radiological contrast agents is feasible and represents an innovative strategy that opens the possibility to fully exploit intraoperative imaging modalities such as CT, PET, and hybrid imaging. Along with imaging contrast agents, EVs could also deliver controlled, high doses of antitumor therapeutic agents directly to cancer cells. This dual capability makes them unique for the development of theranostic agents that can pave the way for improved strategies for early cancer detection and treatment and usher in a new era of precision radiology and nuclear medicine.

## Data Availability

The data presented in this study are available on request from the corresponding author. The data are not publicly available due to privacy issues.

## Supplementary Materials

The following supporting information can be downloaded at: https://www.mdpi.com/article/10.3390/pharmaceutics14122766/s1, Figure S1: Additional cryo-EM images; Figure S2: Evaluation of PDEVs integrity. Figure S3: Immunoblot analysis of TSG101. Table S1: Results of the NTA analysis.
