# Supplementary material for: Increased Sensitivity of Computed Tomography Scan for Neoplastic Tissues Using the Extracellular Vesicle Formulation of the Contrast Agent Iohexol
Source: Pharmaceutics. 2022 Dec 10;14(12):2766. doi: 10.3390/pharmaceutics14122766 (PMC9786056; doi:10.3390/pharmaceutics14122766)
Supplement: Supplementary file 1 [file pharmaceutics-14-02766-s001.zip › Supplementary Figure S1.pdf]

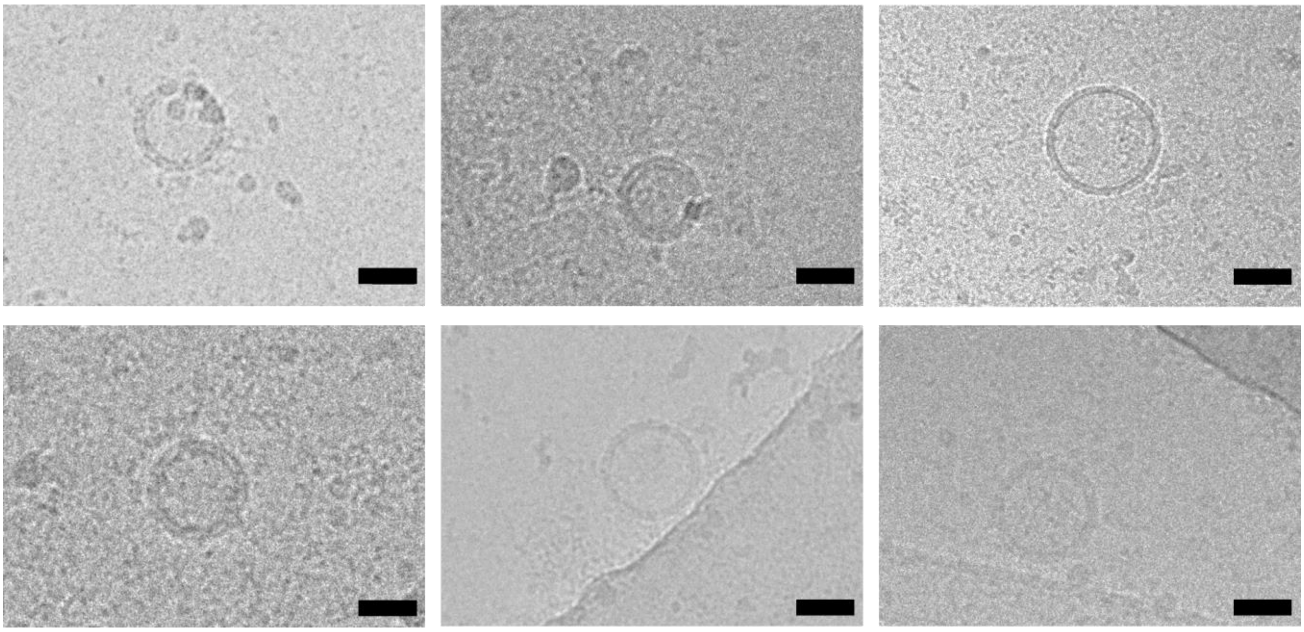

Supplementary Figure S1. Additional cryo-EM images. Representative pictures showing EV morphology and size obtained acquiring 150 field using cryo-electron microscopy, scale bar: 100 nm. Measured sizes are in line with those reported by NTA analysis.
