# Supplementary material for: Increased Sensitivity of Computed Tomography Scan for Neoplastic Tissues Using the Extracellular Vesicle Formulation of the Contrast Agent Iohexol
Source: Pharmaceutics. 2022 Dec 10;14(12):2766. doi: 10.3390/pharmaceutics14122766 (PMC9786056; doi:10.3390/pharmaceutics14122766)
Supplement: Supplementary file 1 [file pharmaceutics-14-02766-s001.zip › Supplementary Figure S2.pdf]

A

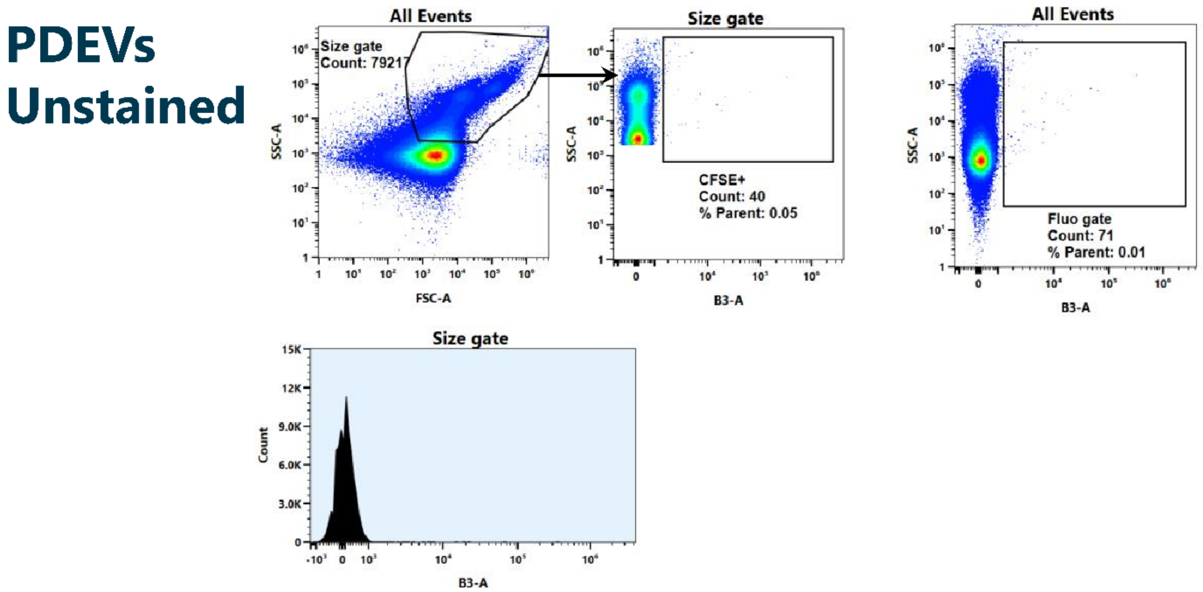


B

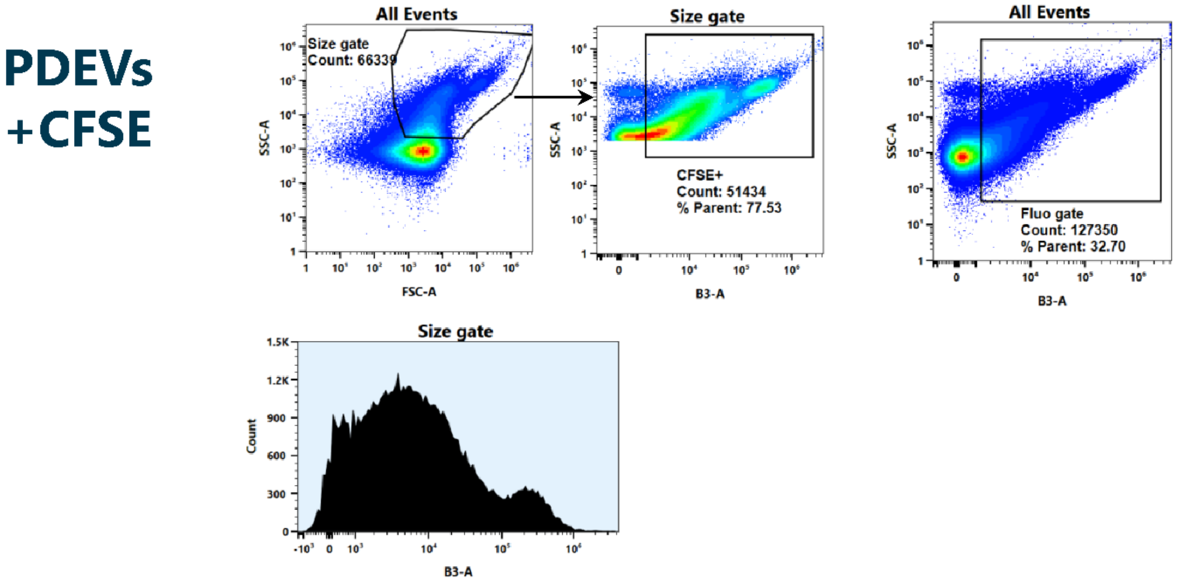


Supplementary Figure S2. Evaluation of PDEVs integrity. A) Flow cytometry analysis of unstained PDEV showing the gating strategy. The cytograms depict: the SSC-A vs FSC-A channels used to trace the Size gate including PDEVs; the SSC-A vs B3-A (green fluorescence triggering) used to trace the CFSE+ gate. B) ) Flow cytometry analysis of CFSE-labeled PDEV showing the % of CFSE positive (CFSE+) vesicles. The cytograms depict: the SSC-A vs FSC-A channels used to trace the Size gate including PDEVs; the SSC-A vs B3-A (green fluorescence triggering) used to trace the CFSE+ gate. CFSE, carboxyfluorescein succinimidyl ester; PDEVs, patient-derived extracellular vesicles.
