# Supplementary material for: Increased Sensitivity of Computed Tomography Scan for Neoplastic Tissues Using the Extracellular Vesicle Formulation of the Contrast Agent Iohexol
Source: Pharmaceutics. 2022 Dec 10;14(12):2766. doi: 10.3390/pharmaceutics14122766 (PMC9786056; doi:10.3390/pharmaceutics14122766)
Supplement: Supplementary file 1 [file pharmaceutics-14-02766-s001.zip › Supplementary Figure S3.pdf]

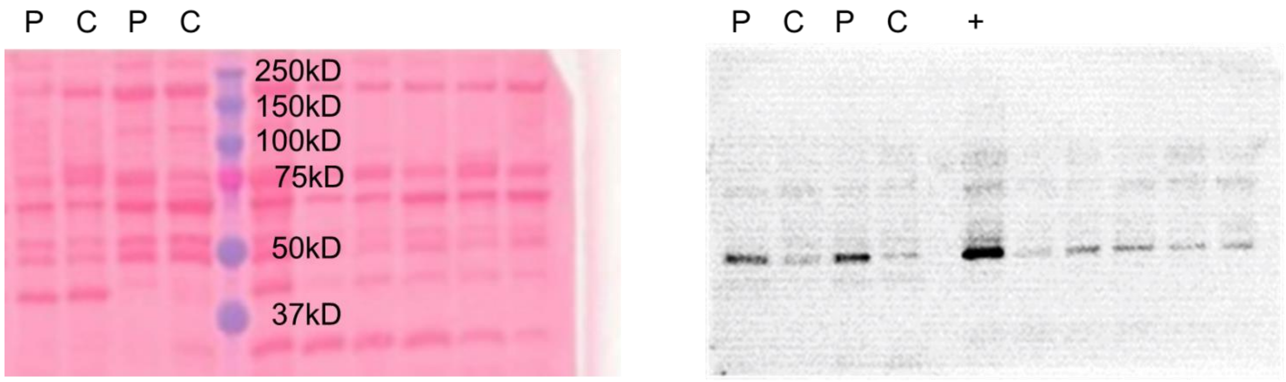

Supplementary Figure S3. Immunoblot analysis of the Tumor Susceptibility Gene-101 (TSG101) expression in plasma-derived EVs from patients (P) or healthy controls (C). +: positive control.
