# Supplementary material for: Increased Sensitivity of Computed Tomography Scan for Neoplastic Tissues Using the Extracellular Vesicle Formulation of the Contrast Agent Iohexol
Source: Pharmaceutics. 2022 Dec 10;14(12):2766. doi: 10.3390/pharmaceutics14122766 (PMC9786056; doi:10.3390/pharmaceutics14122766)
Supplement: Supplementary file 1 [file pharmaceutics-14-02766-s001.zip › Supplementary Table S1.pdf]

Supplementary Table S1. Results of the NTA analysis performed on the PDEV sample used for *in vivo* experiments, showing the size distribution. SD: Standard deviation. D10: diameter (nm) of the particles that is the 10th percentile. D50: diameter (nm) of the particles that is the 50th percentile. D90: diameter (nm) of the particles that is the 90th percentile.

Stats: Mean +/- Standard Error	
Mean:	195.9 +/- 1.4 nm
Mode:	160.3 +/- 12.5 nm
SD:	96.7 +/- 6.5 nm
D10:	109.8 +/- 2.8 nm
D50:	167.3 +/- 6.4 nm
D90:	318.5 +/- 8.3 nm
Concentration:	7.58e+10 +/- 8.42e+09 particles/ml
	40.2 +/- 4.3 particles/frame
	50.2 +/- 5.1 centres/frame
